# Expanding the Donor Pool in Pediatric Transplantations: Successful Domino Liver Transplantation From the Youngest Reported Pediatric DCD Donor Using Normothermic Regional Perfusion in the United States

**DOI:** 10.1111/petr.70498

**Published:** 2026-10-11

**Authors:** Anastasia M. Kahan, Francisca van der Schyff, Alan G. Contreras, Dave N. Peterson, M. Kyle Jensen, Jill Sweney, Lindsey N. Troy, Jean F. Botha, Zachary J. Kastenberg

**Affiliations:** ^1^ Department of Surgery, Division of Pediatric Surgery University of Utah Salt Lake City Utah USA; ^2^ Department of Surgery, Department of Transplant Surgery Intermountain Health Salt Lake City Utah USA; ^3^ Department of Anesthesia University of Utah Salt Lake City Utah USA; ^4^ Department of Pediatrics, Division of Gastroenterology, Hepatology, & Nutrition University of Utah Salt Lake City Utah USA; ^5^ Department of Pediatrics, Division of Critical Care University of Utah Salt Lake City Utah USA

## Abstract

**Introduction:**

Normothermic regional perfusion (NRP) during donation after circulatory death (DCD) improves abdominal organ utilization by limiting warm ischemia time. Its application in young pediatric donors remains exceptionally rare; the youngest previously reported DCD‐NRP donor in the United States was 5 years of age.

**Methods:**

We describe DCD‐NRP organ procurement from a 23‐month‐old donor and the subsequent use of the liver graft in a domino liver transplantation involving two pediatric recipients. The kidneys were recovered en bloc. The donor, supported on veno‐arterial extracorporeal membrane oxygenation (VA‐ECMO) following hypoxic–ischemic injury, underwent DCD‐NRP using the preexisting ECMO cannulation after thoracic exclusion of the cerebral and coronary circulations. The recovered graft was transplanted into a 4‐year‐old with maple syrup urine disease (MSUD), whose explanted native liver was then used as a domino graft for an 18‐month‐old with biliary atresia.

**Results:**

NRP was established rapidly with restoration of metabolic activity, and both transplants were technically successful with good early graft function. The MSUD recipient underwent re‐laparotomy for a suspected intra‐abdominal source of sepsis (no anastomotic leak identified; cultures negative) and recovered with appropriately trending branched‐chain amino acid levels, and was discharged on posttransplant Day 17. The domino recipient had an uncomplicated course and was discharged on posttransplant Day 6.

**Conclusion:**

To our knowledge, this is the youngest DCD‐NRP liver donor reported in the United States and the youngest domino liver transplantation performed using a DCD‐NRP graft. Deliberately pairing NRP with domino transplantation may synergistically expand the critically limited pediatric donor pool.

## 1 Introduction

Normothermic regional perfusion (NRP) in donation after circulatory death (DCD) has emerged as a transformative technique to optimize abdominal organ recovery by mitigating warm ischemia time, thereby improving organ availability [1, 2, 3]. In controlled DCD liver transplantation, NRP‐recovered grafts have been associated with lower rates of ischemic cholangiopathy and primary non‐function and with outcomes comparable to donation after brain death [1]. Reflecting this evidence, the American Society of Transplant Surgeons has endorsed the advancement and broader adoption of NRP and has published standards to guide its safe implementation [2, 3].

Despite this recommendation, NRP applications in the pediatric transplant population remain extremely limited. To date, the youngest reported DCD‐NRP donor in the world was 2.5 months in Spain [4], and the youngest in the US was 5 years old, with the recovered organ transplanted into an adult recipient [5]. The expansion of DCD‐NRP into pediatric populations has yet to be increased or fully adopted in the United States, and would serve to expand the donor population in children who are already at a disadvantage due to long wait times and decreased organ availability [6].

Herein, we report the successful use of DCD‐NRP for liver transplantation from a 23‐month‐old donor, with the liver graft subsequently used in a domino liver transplantation benefiting two separate pediatric recipients. To our knowledge, this is the youngest DCD‐NRP liver donor reported in the United States and the youngest domino liver transplantation performed using a DCD‐NRP graft. We demonstrate the feasibility of expanding the donor pool by applying this technique to the youngest pediatric populations.

## 2 Procedure

### 2.1 Description of the DCD‐NRP Donor

The deceased donor was a 23‐month‐old male (weight 10.9 kg, BMI 19.38 kg/m^2^) with a history of hypertrophic cardiomyopathy complicated by multiple episodes of cardiac arrest. During a third arrest, he underwent extracorporeal cardiopulmonary resuscitation (eCPR) with initiation of veno‐arterial extracorporeal membrane oxygenation (VA‐ECMO) via cervical right internal jugular (RIJ) vein and right common carotid (RCC) artery cannulation. Despite successful cannulation and stabilization on the VA‐ECMO circuit, his clinical course was complicated by extensive hypoxic–ischemic brain injury, with neuroimaging demonstrating large ischemic strokes.

Given the severity of neurologic injury and lack of meaningful recovery, the prognosis was deemed poor by his multidisciplinary care team. After comprehensive discussions with the family, a decision was made to transition to comfort‐focused care. The patient remained on supportive measures for a total of 10 days following this decision, during which time goals of care were reaffirmed. In alignment with the family's wishes and institutional protocols, the patient was subsequently considered for organ donation under donation after circulatory death (DCD), ultimately proceeding to normothermic regional perfusion (NRP) and subsequent abdominal organ retrieval. Terminal donor laboratory evaluation performed approximately 2 h prior to donation demonstrated AST 54 U/L, ALT 12 U/L, alkaline phosphatase 74 U/L, total bilirubin 1.0 mg/dL, and direct bilirubin 0.4 mg/dL, and creatinine of 0.25. Serum albumin was 3.0 g/dL and total protein was 5.0 g/dL.

### 2.2 DCD‐NRP Donor Procedure

Following withdrawal of life‐sustaining therapy in the ICU, the donor underwent circulatory arrest and was transferred to the operating room. He was declared deceased based on standard institutional DCD criteria; the time from extubation to circulatory arrest was 30 min. A mandatory 5‐min no‐touch observation period was subsequently completed, after which death was reconfirmed. Surgical incision occurred 1 min following this second confirmation, after completion of the 5‐min no‐touch period.

A median sternotomy was performed to facilitate control of the thoracic vasculature. The ascending aorta and arch vessels were exposed. The left common carotid artery (LCC) and left subclavian artery (LSC) were identified, dissected, and ligated to prevent cerebral reperfusion. The ascending aortic arch was then cross‐clamped proximal to the origin of the cardiac vessels, ensuring complete exclusion of cardiac and cerebral circulation. The preexisting 14‐French cannula in the RIJ vein and 13‐French cannula in the RCC artery were utilized for rapid initiation of NRP. Cannula positioning was confirmed to be distal to the aortic cross‐clamp, thereby directing oxygenated flow exclusively to the abdominal organs while maintaining complete exclusion of the heart and brain in accordance with ethical and legal standards for DCD organ recovery at the procuring institution.

Time from withdrawal to initiation of NRP was 36 min. Time from circulatory death to initiation of NRP was 6 min. Abdominal NRP was maintained for 103 min, with pump flow sustained between 1.7 and 2.1 L/min throughout the perfusion period. Adequate perfusion was confirmed by normalization of hemodynamic parameters within the circuit, improvement in liver coloration, and restoration of metabolic activity as assessed by serial blood gas analysis and lactate clearance. Serial metabolic assessments documented progressive improvement during NRP, with lactate declining from 12.4 mmol/L at initiation to 9.9 mmol/L at cross‐clamp and arterial pH increasing from 7.02 to 7.6.

Following completion of the NRP phase, standard abdominal organ procurement was performed. In situ aortic flush was performed with 5000 mL of University of Wisconsin (UW) solution, followed by back table portal flush with 1000 mL UW solution. There was a small right accessory hepatic artery. The kidneys were recovered en bloc and separately allocated, with both kidneys transplanted into an adult recipient. Both grafts were functioning at last available follow‐up with a most recent creatinine of 0.8 mg/dL. Thus, all three abdominal organs recovered from the pediatric DCD donor were successfully transplanted.

#### 2.2.1 Recipient 1, DCD‐NRP Liver Graft

The recipient of the DCD‐NRP liver graft was a 4‐year‐old female (weight 16.9 kg, BMI 19.13 kg/m^2^) with a diagnosis of maple syrup urine disease (MSUD) managed medically since infancy. Her clinical course was characterized by stringent dietary restrictions, supplemented by metabolic formula providing approximately 1 g of protein per day. Despite adherence to dietary management, she remained at ongoing risk for metabolic decompensation. Given the burden of lifelong dietary restriction and risk of recurrent metabolic crises, she was evaluated and accepted as an appropriate candidate for liver transplantation as definitive therapy. At the time of transplant, she was metabolically stable on her prescribed dietary regimen, with no evidence of acute decompensation.

She subsequently underwent deceased‐donor whole‐graft liver transplantation with bicaval replacement and duct‐to‐duct biliary reconstruction and, in the same operative event, served as the domino donor. During the recipient hepatectomy, the native liver was explanted with deliberate preservation of the hepatic arterial branches until final removal to ensure its suitability as a domino graft. After heparinization, the liver was removed including the retrohepatic inferior vena cava and was passed off for back‐table flushing and preparation as the domino graft. The allograft was then implanted with a with bicaval replacement followed by an end‐to‐end portal vein anastomosis and the graft was reperfused without significant hemodynamic instability. Arterial reconstruction was done from donor common hepatic artery into the recipient common hepatic artery. Intraoperative Doppler ultrasound confirmed appropriate directional flow and waveforms in the hepatic artery, portal vein, and hepatic venous outflow, Finally, a duct‐to‐duct biliary reconstruction was performed. A single right‐upper‐quadrant drain was placed prior to closure.

#### 2.2.2 Recipient 2, Domino Liver Graft

The liver graft from the MSUD donor was prepared on the back table. A segment of an iliac vein from a deceased donor was anastomosed around the suprahepatic cava as an extension. The recipient was an 18‐month‐old child (weight 12.3 kg, BMI 17.62 kg/m^2^) with a diagnosis of biliary atresia (BA) who had previously undergone Kasai portoenterostomy at 78 days of life. Despite initial surgical management, the patient developed progressive liver disease with recurrent episodes of cholangitis consistent with non‐functioning Kasai. The patient also had evidence of portal hypertension, including known esophageal varices. PELD was calculated as 6. Given ongoing hepatic dysfunction and complications from her previously performed Kasai, the patient was evaluated and accepted as an appropriate candidate for liver transplantation.

The domino graft was implanted using a bicaval technique as described above. The arterial reconstruction was tailored to the graft's anatomy, which included a replaced left hepatic artery. The recipient, with a replaced right hepatic artery and a large left hepatic artery arising from an anatomic common hepatic artery, and a smaller accessory left hepatic artery, had multiple arterial targets. The recipient's dominant left hepatic artery was anastomosed to the donor right hepatic artery and the recipient's accessory left hepatic artery to the donor's replaced left hepatic artery. Intraoperative Doppler ultrasound confirmed appropriate directional flow and waveforms in the hepatic artery, portal vein, and hepatic venous outflow. Biliary continuity was restored with a hepaticojejunostomy to the existing Roux limb, and a single right‐upper‐quadrant drain was placed prior to closure.

### 2.3 Postoperative Course and Outcomes

#### 2.3.1 Recipient 1, DCD‐NRP Liver Graft

The early course was uncomplicated, with down‐trending transaminases and transfer from the intensive care unit on posttransplant (+) Day 1. Beginning on +6, she developed a febrile episode followed over the subsequent 48 h by progressive clinical deterioration—persistently poor enteral intake, abdominal pain, nausea, tachycardia, leukocytosis, and new large‐volume ascites with an increasing oxygen requirement. On +9 she underwent exploratory laparotomy with washout and temporary open‐abdomen management for suspected intra‐abdominal infection, followed by a second washout, abdominal closure, and chest‐tube placement the following day; no anastomotic leak was identified and cultures remained negative. Her postoperative course was further complicated by acute kidney injury and a right‐predominant pleural effusion, managed with diuresis, albumin‐based volume replacement, and broadened empiric antimicrobial coverage, with immunosuppression briefly held and then resumed on a renal‐sparing regimen. She was extubated to room air, advanced to goal enteral and oral nutrition, and demonstrated appropriately trending branched‐chain amino acid levels on serial metabolic monitoring, consistent with correction of her underlying metabolic defect. She was discharged home on +17 (postoperative day 8 from the index re‐laparotomy) on a liberalized diet and in excellent condition.

#### 2.3.2 Recipient 2, Living Donor Whole Liver Graft

The early postoperative course was complicated by coronavirus‐associated pneumonia/bronchiolitis requiring high‐flow nasal cannula, which was weaned to room air, and was treated with broad‐spectrum antibiotics. Graft function was excellent, with progressively normalizing liver enzymes; her diet was advanced to a regular intake, surgical drains were removed without complication, and she was transferred from the intensive care unit to the floor on +2. She had intermittent hypertension that resolved with as‐needed antihypertensives, and immunosuppression was established with tacrolimus and a methylprednisolone taper alongside fluconazole and aspirin prophylaxis. She was discharged to home on +6.

## 3 Discussion

To our knowledge, this is the youngest DCD‐NRP liver donor reported in the United States to date, and the youngest domino liver transplantation performed using a DCD‐NRP graft. This case demonstrates that NRP can be technically accomplished in a very small donor. Notably, preexisting VA‐ECMO cannulation was repurposed for regional perfusion after thoracic exclusion of the cerebral and coronary circulations, allowing rapid, controlled normothermic reperfusion of the abdominal organs in accordance with established standards of practice [2, 3]. Pediatric donor organs are scarce, and strategies that increase the number and quality of usable grafts have a disproportionate impact for children awaiting transplantation [6, 7, 8]. In controlled DCD liver transplantation, NRP has been associated with improved graft utilization and with outcomes comparable to donation after brain death, including reductions in ischemic cholangiopathy and primary non‐function [1, 8]. Extending NRP to the youngest pediatric donors, as in the present case, may meaningfully enlarge the size‐ and age‐matched donor pool for pediatric recipients. Because young children face disproportionate waitlist mortality, driven in large part by the shortage of size‐matched grafts and the diversion of otherwise suitable pediatric livers to adult recipients [6], even incremental expansion of the youngest‐donor pool through techniques such as DCD‐NRP has the potential to convert previously unrealized donations into life‐saving transplants and to reduce preventable deaths among children awaiting liver transplantation.

There are important ethical considerations to account for in NRP in pediatric donation which extend beyond those of adult donation and warrant explicit attention [9]. The central point of concern is preservation of the dead donor rule: [10, 11] in thoracoabdominal and abdominal NRP this is achieved through a mandatory hands‐off observation period before procurement begins and through complete isolation of the cerebral circulation, in this case, by ligation of the arch vessels and confirmation that perfusion is confined distal to the aortic cross‐clamp with use of preexisting ECMO cannulation to assist in rapid perfusion in an isolated circuit. More broadly in children being evaluated for DCD‐NRP, these safeguards must be paired with heightened attention to family‐centered consent, transparency, and public trust. Adherence to these principles is what allows the donor‐pool benefits of pediatric NRP to be realized without compromising ethical or legal standards.

The principal contribution of this case is to show how two independent strategies, DCD‐NRP and domino transplantation, can be used synergistically to expand the donor pool in very young children. NRP addresses the supply side of this problem by improving both the utilization and the quality of pediatric DCD liver grafts [1, 2]. Domino transplantation effectively converts one transplant into two [12, 13]. Published pediatric and mixed series confirm that this is done safely, with normalization of amino acid levels in the MSUD recipient, tolerance of an unrestricted protein diet, and no de novo MSUD in the domino recipient [12, 13]. In the present case these mechanisms compounded: a single circulatory‐death donation, rendered transplantable through NRP, ultimately provided definitive therapy for two children. As pediatric NRP matures, deliberately pairing it with domino pathways may represent a concrete, repeatable way to multiply the yield of each scarce pediatric liver donation.

This case also highlights an important disparity in current OPTN allocation policy as it pertains to pediatric donors: under current OPTN policy (policy 9) [14], livers procured from pediatric brain‐dead donors carry explicit pediatric allocation priority: organs are offered first to compatible pediatric candidates within a defined geographic radius before adult candidates are considered. There is no equivalent policy for pediatric circulatory‐death donors. A DCD liver allocation does not distinguish the recipient age based on the donor classification, meaning a liver procured from a pediatric DCD may be allocated to an adult before a waiting pediatric patient, as was seen in the previously reported youngest USA experience [5]. This asymmetry is clinically significant. Pediatric waitlist mortality is driven in large part by the scarcity of size‐ and age‐matched grafts, and the diversion of suitable pediatric livers to adult recipients has been identified as a contributor to preventable deaths in children awaiting transplantation. If DCD‐NRP is increasingly applied to expand the pediatric donor pool, the absence of pediatric priority in DCD allocation becomes a more pressing concern, and the present case illustrates this directly: a 23‐month‐old DCD donor whose organs were ultimately used to benefit two pediatric recipients was not, at the time of allocation, subject to any policy mechanisms that would ensure the pediatric candidates received preferential access. While in this instance the clinical team identified an appropriate pediatric use, there is no systematic policy safeguard to ensure the same outcome in future cases. This case highlights that there should be a discussion at a national level advocating for policy change that provides children with priority when it comes to the allocation of pediatric DCD organs. Closing this gap would ensure that the expanding pool of pediatric DCD organs, increasingly recoverable through techniques such as NRP, is allocated in a manner that reflects the ethical imperative that young donors preferentially serve young patients.

## 4 Conclusion

Herein we report the youngest DCD‐NRP liver donor described in the United States to date, with the 23‐month‐old graft used in a domino liver transplantation that supported two pediatric recipients. This experience demonstrates that NRP can be applied in the youngest pediatric DCD donors and, when combined with domino transplantation, offers a strategy to expand the critically limited pediatric donor pool.

## Funding

The authors have nothing to report.

## Conflicts of Interest

The authors declare no conflicts of interest.

## Data Availability

The data that support the findings of this study are available from the corresponding author upon reasonable request.
